# NSOM/QD-Based Direct Visualization of CD3-Induced and CD28-Enhanced Nanospatial Coclustering of TCR and Coreceptor in Nanodomains in T Cell Activation

**DOI:** 10.1371/journal.pone.0005945

**Published:** 2009-06-17

**Authors:** Liyun Zhong, Gucheng Zeng, Xiaoxu Lu, Richard C. Wang, Guangming Gong, Lin Yan, Dan Huang, Zheng W. Chen

**Affiliations:** 1 Department of Microbiology and Immunology, Center for Primate Biomedical Research, University of Illinois College of Medicine, Chicago, Illinois, United States of America; 2 School for Information and Optoelectronic Engineering, South China Normal University, Guangzhou, Guangdong, China; New York University School of Medicine, United States of America

## Abstract

Direct molecular imaging of nano-spatial relationship between T cell receptor (TCR)/CD3 and CD4 or CD8 co-receptor before and after activation of a primary T cell has not been reported. We have recently innovated application of near-field scanning optical microscopy (NSOM) and immune-labeling quantum dots (QD) to image Ag-specific TCR response during in vivo clonal expansion, and now up-graded the NSOM/QD-based nanotechnology through dipole-polarization and dual-color imaging. Using this imaging system scanning cell-membrane molecules at a best-optical lateral resolution, we demonstrated that CD3, CD4 or CD8 molecules were distinctly distributed as single QD-bound molecules or nano-clusters equivalent to 2–4 QD fluorescence-intensity/size on cell-membrane of un-stimulated primary T cells, and ∼6–10% of CD3 were co-clustering with CD4 or CD8 as 70–110 nm nano-clusters without forming nano-domains. The ligation of TCR/CD3 on CD4 or CD8 T cells led to CD3 nanoscale co-clustering or interaction with CD4 or CD8 co-receptors forming 200–500 nm nano-domains or >500 nm micro-domains. Such nano-spatial co-clustering of CD3 and CD4 or CD3 and CD8 appeared to be an intrinsic event of TCR/CD3 ligation, not purely limited to MHC engagement, and be driven by Lck phosphorylation. Importantly, CD28 co-stimulation remarkably enhanced TCR/CD3 nanoscale co-clustering or interaction with CD4 co-receptor within nano- or micro-domains on the membrane. In contrast, CD28 co-stimulation did not enhance CD8 clustering or CD3–CD8 co-clustering in nano-domains although it increased molecular number and density of CD3 clustering in the enlarged nano-domains. These nanoscale findings provide new insights into TCR/CD3 interaction with CD4 or CD8 co-receptor in T-cell activation.

## Introduction

While T cell receptors (TCR) recognize major histocompatibility complex (MHC)-associated peptide complex (MHCp) [1], [2], [3], [4], CD4 or CD8 co-receptor increases the sensitivity of TCR recognition and thus facilitates TCR/CD3-mediated signaling and T cell activation [5], [6], [7], [8]. The enhanced TCR recognition of MHCp is attributed to the ability of CD4 or CD8 co-receptor to bind to non-polymorphic regions of MHC I or II molecule, and facilitate TCR-MHCp interaction [6], [9], [10]. On the other hand, CD4 or CD8 is associated with the kinase Lck, thus CD4 or CD8 binding to MHC recruits Lck close to the TCR/CD3 and helps to phosphorylate TCR/CD3 complex for initiation of CD3-induced T cell signaling and activation [5]. However, precise mechanisms by which interaction between TCR/CD3 and CD4 or CD8 co-receptor is sustained and regulated are poorly understood.

Direct molecular imaging of nano-spatial relationship between TCR/CD3 and CD4 or CD8 co-receptor on a primary T cell has not been reported. Scanning fluorescence nanoscale visualization of each of these molecules on cell-surface of CD4 or CD8 T cells is indeed lacking. Early studies of TCR/CD3-CD4 co-localization or interaction using conventional techniques such as flow cytometry, fluorescence/confocal microscopy or indirect biochemistry analyses of membrane rafts appear to be inconclusive with conflicting results [11], [12], [13], [14]. Although recent studies using microscopy fluorescence resonance energy transfer (FRET) have demonstrated that MHCp engagement of TCR on transfected T hybridoma cells can induce TCR/CD3 interaction (proximity) with CD4 or CD8 co-receptor in the immunologic synapse [15], [16], a sustained interaction between TCR/CD3 and co-receptor for achieving or maintaining full T-cell activation has not been directly imaged at nanoscale. In this context, it is not known whether the TCR/CD3-CD4 (CD8) interaction as demonstrated by the FRET can also be the intrinsic capability of the TCR/CD3 activation pathway, not purely limited to the MHC engagement. Such intrinsic capability of TCR/CD3 to recruit CD4 or CD8 co-receptor would implicate a positive-feedback or self-enhancing mechanism since strengthening CD3-CD4 interaction upon TCR/CD3 activation would promote Lck-ZAP70 phosphorylation and sustain down-stream signaling/activation. Furthermore, the possibility that CD28 co-signaling can enhance potential TCR/CD3 interaction with CD4 co-receptor for full T cell activation has not been addressed, although CD28 co-stimulation has been shown to potentiate T cell activation [15], [16], [17], [18], [19], [20], [21], [22], [23], [24], [25], [26], [27], [28], [29], [30], [31], [32], [33]. We presume that these fundamental questions can be addressed using a best-optical-resolution nanoscale imaging system for direct molecular visualization of TCR/CD3 interaction with CD4 or CD8 co-receptor during T cell activation.

We have recently innovated the use of near-field scanning optical microscopy [NSOM [17], [18], [19], [20], [21], [22]] and quantum dots [QD [23]] for nanoscale imaging of antigen-specific TCR molecules on T-cell surface [24]. Our novel NSOM/QD-based nanoscale imaging [24], [25], [26], [27] overcomes the outstanding problem of photobleaching conventional immune-fluorochromes [28], [29], [30] while executing near-field imaging that breaks-through the diffraction limit, and provides highly-reproducible fluorescence imaging with a best optical resolution of ∼50 nm. In fact, our NSOM/QD-based nanoscale fluorescence imaging clearly reveals that single-QD-bound TCR undergo nano-spatial re-distribution to array and form high-density TCR nanoclusters or nanodomains during an *in vivo* clonal expansion [24]. In the current study, we have up-graded the NSOM/QD-based nanotechnology through dipole-polarization and dual-color imaging to visualize nanoscale profiles for distribution and organization of CD3, CD4 or CD8, as well as nano-spatial relationship between CD3 and CD4 or CD8 on cell-membrane. The NSOM/QD-based imaging also demonstrates that TCR/CD3 activation can intrinsically induce nanoscale CD3 co-clustering or interaction with CD4 or CD8 co-receptors, which forms 200–500 nm nano-domains or >500 nm micro-domains. More importantly, the NSOM/QD-based imaging shows that CD28 co-stimulation can remarkably enhance TCR/CD3 nanoscale co-clustering or interaction with CD4, but not CD8, co-receptor within nano- or micro-domains on the membrane.

## Results

### CD3, CD4 or CD8 molecules were distinctly distributed as single QD-bound molecules or nano-clusters on cell-membrane of un-stimulated primary T cells

As an initial effort to directly visualize TCR/CD3 co-clustering or interaction with CD4 or CD8 co-receptor during T cell activation, we first imaged nanoscale distribution or relationship of each of these molecules on cell-surface of un-stimulated primary T cells using the NSOM/QD-based polarized imaging system. This polarized imaging system simultaneously collects two polarization components of images [0° and 90° polarization components, [25]]. The polarized detection of one QD as we previously published [25] allowed us to judge single or multiple QD-bound CD3, CD4 or CD8 molecules on cell-membrane. To facilitate evaluation of nano-spatial distribution and organization of CD3, CD4 or CD8, nanostructures of these molecules were defined based on the nano-concept and immune-fluorescence dot sizes of them: (i) nanoclusters, ≤200 nm, (ii) nano-domains, >200 but <500 nm, and (iii) micro-domains, ≥500, as we recently described [24]. Interestingly, on cell-surface of un-stimulated CD4 or CD8 T cells, ∼5–8% of CD3 were single QD-bound molecules (Supporting information, Figure S1Ai, Figure S1Aii); most of CD3 molecules were detected as nano-clusters equivalent to 2–4 QD fluorescence-intensity and size (i.e. full width at half maximum (FWHM)) (Supporting information, Figure S1Aii). However, these fluorescence QD dots representing potential monomers, dimers/multimers, or pre-clustering of CD3/TCR [31] were clearly distinct with ≥40–50 nm distance from each other under the high-resolution NSOM. Like CD3 molecules, CD4 or CD8 co-receptors were similarly distributed on cell-surface of un-stimulated T cells (Supporting information, Figure S1B, Figure S1C), although more single QD-bound CD8 co-receptors were seen on the membrane than CD3 and CD4. It was noteworthy that no significant fluorescence was observed when T cells were stained with control isotype Ab followed by conjugated QD, or QD only (Figure S1D), or isotype Ab only (Figure S1E), suggesting the specificity of the antibody and QD staining.

**Figure 1:**
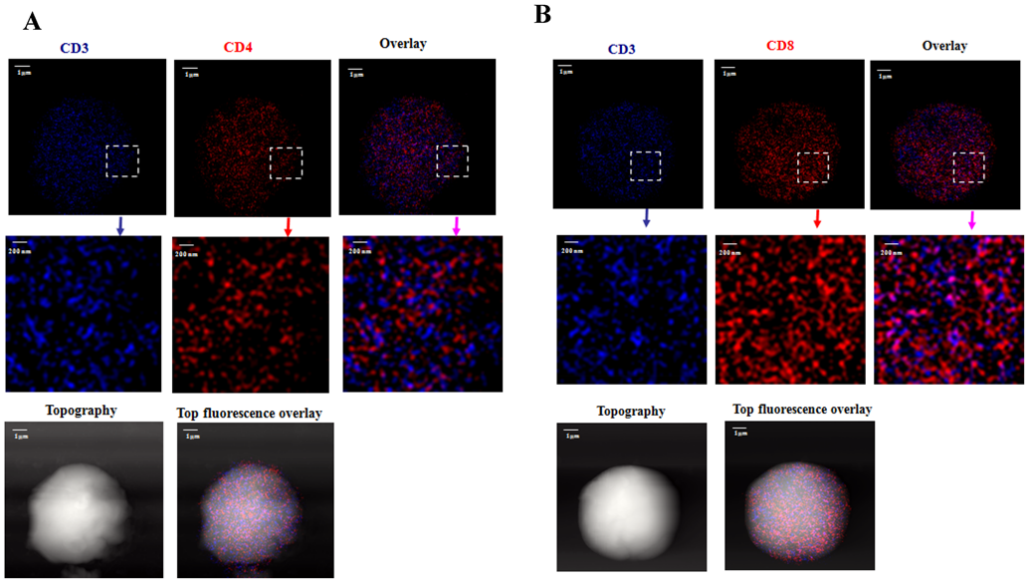
The NSOM/QD-based two-color imaging showed that about 6–10% of CD3 were co-clustering with CD4 or CD8 as 70–110 nm nano-clusters. Upper panels in each of the sub-figures show fluorescence images of a whole cell; Middle panels show zoom images of the areas as indicated by the squares on the top panels. Lower panels show the T cell topography and topography-fluorescence overlay images. Scale bars are indicated in each sub-figure. The integration time for all the images was 30 ms with 400*400 scanning lines. (A) NSOM/QD-based two-color images of one representative of the un-stimulated CD4 T-cells. Majority of CD3 and CD4 were distinctly distributed on the membrane; about 6–10% of CD3 were co-localizing with CD4 as 80–100 nm nanoclusters. Of note, these co-localized nanoclusters did not form nano- or micro-domains. (B) NSOM/QD-based two-color images of one representative of the un-stimulated CD8 T-cells. The nanoscale distribution pattern was similar to what was seen for CD4 T cells.

We then utilized the NSOM/QD-based dual-color nanoscale imaging system to visualize nano-spatial relationship between CD3 and CD4 or CD8 on cell-membrane of un-stimulated primary T cells. Most of detectable CD3 were distributed distinctly (≥40–50 nm distance) away from CD4 or CD8 co-receptor on membrane of CD4 or CD8 T cells (Fig. 1A, 1B). Interestingly, about 6–10% of CD3 co-localized with CD4 or CD8 as ∼70–110 nm nanoclusters on cell-surface of un-stimulated T cells (Fig. 1A, 1B, Figure S2A, Figure S2B, Figure S2C, Figure S2D). However, these co-localized CD3 and CD4 or CD8 remained individually distinct without forming any advanced nanostructures such as nano-domains (Fig. 1A, 1B). Thus, the NSOM/QD-based nanoscale imaging studies of un-stimulated primary T cells demonstrated for the first time that even without MHCp stimulation or TCR ligation, small numbers of CD3 co-localized with CD4 or CD8 as 70–110 nm nanoclusters on cell-surface whereas most of them display distinct nano-clusters equivalent to 1–4 QD-bound molecules. These findings made it possible to image nano-spatial re-distribution or re-organization of TCR/CD3 and CD4 or CD8 co-receptor during T cell activation.

### NSOM/QD-based nanoscale imaging directly visualized CD3 co-clustering or interaction with CD4 co-receptor

**Figure 2:**
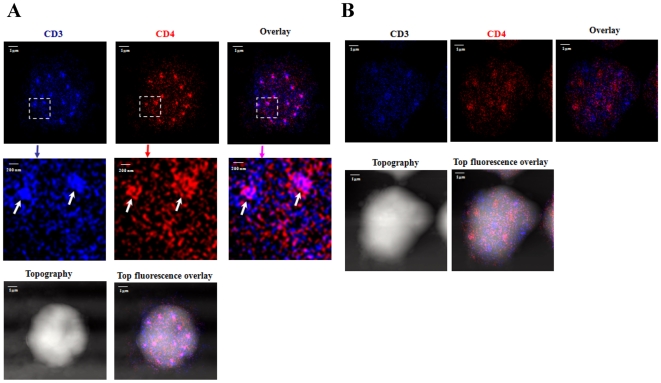
NSOM/QD-based nanoscale two-color imaging directly imaged CD3 co-clustering or interaction with CD4 co-receptor after activation of CD4 T cells by anti-CD3 Ab. The integration time for all the images was 30 ms with 400*400 scanning lines. (A) The NSOM dual color images of one representative of the anti-CD3 Ab-stimulated T-cells. The upper (whole-cell images) and middle (zoom images) panels show nano-clustering of CD3 (dark blue) and CD4 (red) as well as co-clustering of CD3-CD4 (overlay pink) forming nano-domains or micro-domains on cell surface of an activated T-cell, as illustrated by white arrows. Note that CD3, CD4 or CD3–CD4 nano-domains were hardly seen in un-stimulated T cells. The activated CD4 T cells also had a number of CD3–CD4 co-localized nano-clusters 70–110 nm outside the nano-domains as illustrated by yellow arrows. Lower panels show the T cell topography and topography-fluorescence overlay images. (B) The NSOM dual-color images of one representative of the PMA/Ionomycin-stimulated T-cells. Note that although PMA/Ionomycin stimulation led to the formation of independent CD3 or CD4 nano-domains, there was no apparent co-clustering or co-localization of CD3 and CD4 in the nano-domains on the membrane of PMA/Ionomycin-stimulated T-cells. Lower panels show the T cell topography and topography-fluorescence overlay images.

**Figure 3:**
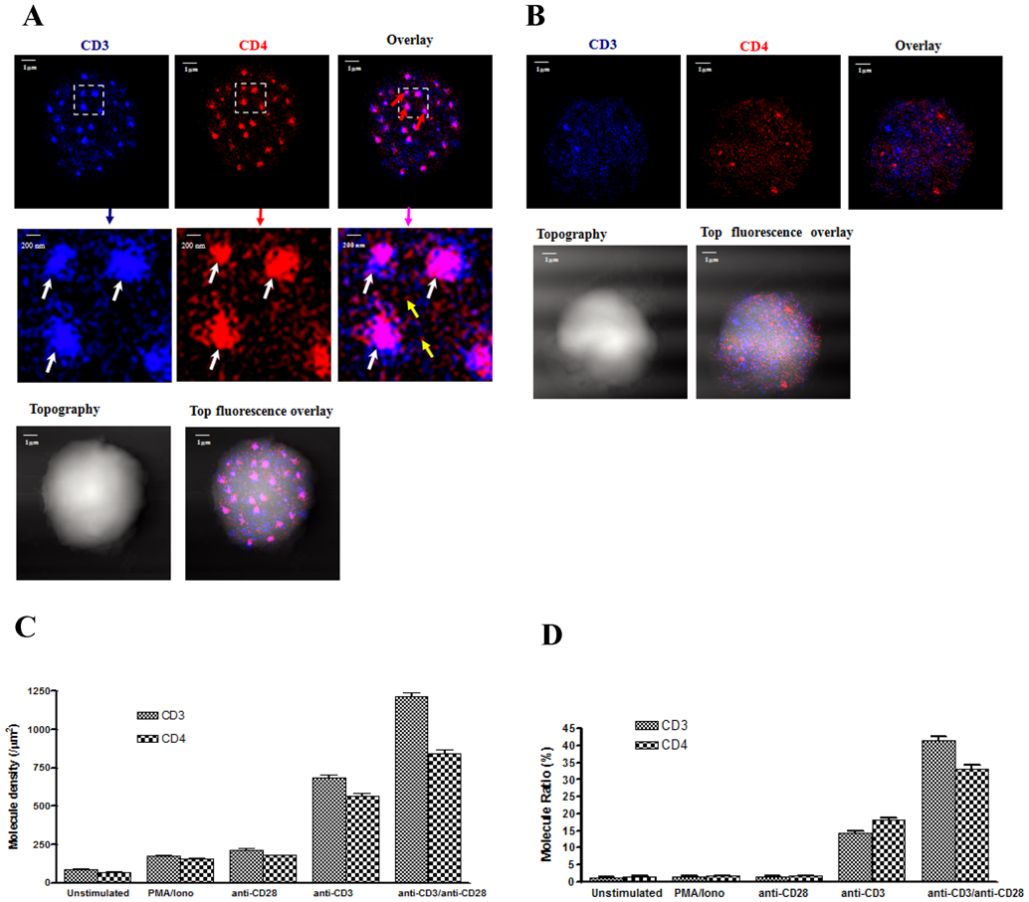
CD28 co-activation remarkably enhanced TCR/CD3 co-clustering or interaction with CD4 on the cell membrane of the anti-CD3 Ab-activated CD4 T cells. (A) The NSOM dual color images of one representative of the T-cells co-stimulated with anti-CD3 Ab and anti-CD28 Ab. The CD28 co-stimulation led to marked increases in numbers of CD3-CD4 co-clustering nano-domains or micro-domains (upper panel) as well as in the density and size (middle panel) of co-localized CD3 and CD4 nano-domains or micro-domains compared to the anti-CD3 Ab alone (Fig. 2A). Lower panels show the T cell topography and topography-fluorescence overlay images. Note that complete co-clustering of CD3 and CD4 took place in the central areas of nano-domains or micro-domains, leaving only small numbers of CD3-only or CD4-only clusters in the peripheral area. CD28 co-stimulation led to increases in secretion of IL-2 by CD3 Ab-activated T cells (data not shown here). (B) The NSOM dual-color images of one representative of the anti-CD28 Ab alone-stimulated T-cells. Note that anti-CD28 Ab stimulation led to the formation of few independent CD3 or CD4 nano-domains, but there was no apparent co-clustering or co-localization of CD3 and CD4 in the nano-domains on the membrane. Lower panels show the T cell topography and topography-fluorescence overlay images. (C) Molecular-density analysis shows the molecular density of CD3 or CD4 molecules on the membrane of un-stimulated T cells, T cells stimulated with anti-CD28 Ab only, anti-CD3 Ab only and T cells co-stimulated with anti-CD3 Ab and anti-CD28 Ab. Anti-CD3 Ab-stimulated T cells exhibited significantly-greater molecular density of CD3 or CD4 compared to unstimulated T-cell, PMA/Ionomycin-stimulated T-cells or anti-CD28 Ab only-stimulated T cells (*p*<0.05); Anti-CD28/anti-CD3 Ab co-stimulation significantly increases density of co-localized CD3–CD4 nano- or micro-domains (*p*<0.05) compared to anti-CD3 Ab stimulation only. Similar results were observed regardless of whether CD3 and CD4 were stained with QD605 or QD655. Up to 10 cells were analyzed. (D) Molecular-number analysis shows the percentages of total CD3 or CD4 molecules that arrayed to form nano- or micro-domains on the membrane of un-stimulated T cells, T cells stimulated with anti-CD28 Ab only, anti-CD3 Ab only and T cells co-stimulated with anti-CD3 Ab and anti-CD28 Ab. Anti-CD3 Ab-stimulated T cells exhibited significantly higher numbers of CD3 or CD4 nano-domains and co-localized CD3–CD4 nanodomains compared to unstimulated T cells, PMA/Ionomycin-stimulated T-cells or anti-CD28 Ab only-stimulated T cells (*p*<0.05); Anti-CD28/anti-CD3 Ab co-stimulation significantly increases numbers of co-localized CD3–CD4 nano- or micro-domains (*p*<0.05) compared to anti-CD3 Ab stimulation only. Up to 10 cells were analyzed.

Although it has been well accepted that CD4 function as co-receptor stabilizing MHCp-TCR/CD3 interaction for promoting TCR signaling, a sustained interaction between TCR/CD3 and co-receptor for achieving or maintaining full T-cell activation has not been directly imaged at nanoscale. One of imaging features for TCR-mediated T cell activation is that TCR/CD3 cluster [32] at the center of the T-cell/antigen presentation cell (APC) interface [33], [34]. Our recent work has also demonstrated that Ag-specific γδ TCR are nano-clustering to form nano-doamains or micro-domains during Ag-induced activation of T cells [24]. We therefore took advantage of TCR/CD3 clustering as a readout of T cell activation to visualize TCR/CD3 co-clustering or interaction with CD4 co-receptor after ligation of TCR/CD3 by anti-CD3 Ab. Interestingly, after the anti-CD3 Ab stimulation of CD4 T cells, CD3 and CD4 both underwent nano-spatial re-distribution, clustered and formed well-organized 200–500 nm nano-domains on cell-surface (Figure S3A, Figure S3B). The percentages of CD3 and CD4 molecules that arrayed to form nano-domains increased significantly to 15.4±4.8% and 20.9±3.7% from 1.13±0.36% and 1.32±0.54%, respectively (Fig. 2, Fig. 3D). The average molecule densities of CD3 and CD4 in nano-domains were 735±22/µm^2^ and 624±30/µm^2^, respectively, which were statistically higher than the average density of CD3 or CD4 clusters observed in un-stimulated T-cells (Fig. 2, Fig. 3C). Importantly, merge NSOM images showed that CD3 and CD4 were co-clustering within the same nano-domains (Fig. 2A), suggesting that the TCR/CD3 interaction with CD4 co-receptor after anti-CD3 Ab stimulation consequently formed co-clustering nanostructures for sustained T cell activation. Consistently, significant production of IL-2 in culture supernatant was detected upon anti-CD3 Ab sitmulation (data not shown).

To determine whether TCR/CD3 interaction or co-clustering with CD4 co-receptor after T cell interaction is an intrinsic event specific for TCR/CD3-mediated signaling and activation, CD4 T cells were stimulated by PMA/Ionomycin (signaling through the inositol phosphates (IP) pathway not CD3) alone without anti-CD3 Ab co-treatment. PMA/Ionomycin control did not induce visible CD3–CD4 co-clustering in nano-domains or micro-domains, although the stimulated T cells got enlarged and developed independent CD3 or CD4 nano-clusters on the membrane (Fig. 2B, 3C and Figure S3C, Figure S3D). This was consistent with the current notion that PMA/Ionomycin induces T-cell activation by a CD3/TCR-indepedent signaling pathway. Furthermore, to explore the potential mechanism underlying the co-clustering of CD3 and CD4, we pre-treated the T cells using Lck inhibitor, 4-Amino-5-(4-chlorophenyl)-7-(t-butyl)pyrazolo[3,4-d]pyrimidine(PP2). Interestingly, the PP2 pre-treatment of T cells greatly reduced the formation of CD4 clusters and CD4/CD3 co-clusters (Figure S4A), suggesting that tyrosine phosphorylation of Lck triggers or drives nanoscale co-clustering or interaction between TCR and co-receptor during CD3-induced T cell activation. Collectively, for the first time the NSOM/QD-based system made it possible to directly image CD3 co-clustering or interaction with CD4 co-receptors in nano-domains or micro-domains after T cell activation, and such nano-spatial CD3–CD4 co-clustering appeared to be an intrinsic event of TCR/CD3 activation without dependence upon MHC.

### CD28 co-stimulation enhanced TCR/CD3 co-clustering with CD4 in the nano-domains or micro-domains

Although CD28 co-stimulation has been shown to enhance TCR/CD3-mediated signaling and activation [35], [36], [37], [38], [39], [40], the possibility that CD28-enhanced T-cell activation involves a stronger interaction between TCR/CD3 and CD4 co-receptor has not been tested. We therefore asked a fundamental question as to whether CD28 co-stimulation could augment the TCR/CD3 nanoscale co-clustering or interaction with CD4 co-receptor in the nano-domains, and therefore enhance CD3-induced CD4 T-cell activation. Thus, CD4 T cells were stimulated with both anti-CD3 Ab and anti-CD28 Ab, and imaged for magnitudes of CD3-CD4 co-clustering in nano-domains or micro-domains. Surprisingly, anti-CD28 Ab co-stimulation remarkably increased anti-CD3 Ab-induced CD3-CD4 co-clustering or interaction, as fluorescence size and density of CD3 and CD4 clusters or CD3-CD4 co-clusters in the co-localized nano-domains and micro-domains were apparently greater than those induced by anti-CD3 Ab stimulation alone (Figs. 3A, 3C, 3D, Figure S5A, Figure S5B). The average molecule densities of CD3 and CD4 in nano- or micro-domains of co-stimulated T cells were 1230±31/µm^2^ and 987±26/µm^2^, respectively, which was statistically significant greater than those of the anti-CD3-stimulated T-cells (Fig. 3C). The CD28 co-stimulation-enhanced CD3 co-clustering with CD4 was consistent with the co-stimulation-mediated increases in expression of IL-2 by CD3 Ab-activated T cells (data not shown here). Interestingly, even though a fraction of CD4 did not co-localize with CD3 in nanodomains/microdomains, they intended to localize themselves close to CD3 (as shown by the yellow arrow heads in Fig. 3A), possibly reflecting CD4 trafficking into and out of TCR/CD3 complex during sustained T-cell activation. In contrast, anti-CD28 Ab stimulation alone did not induce nano-spatial CD3-CD4 co-clustering or interaction (Fig. 3B, Figure S5C, Figure S5D). Thus, these results provided evidence demonstrating that CD28 co-stimulation remarkably enhanced TCR/CD3 co-clustering or interaction with CD4 in the nano-domains or micro-domains on the cell membrane of the anti-CD3 Ab-activated CD4 T cells.

### TCR/CD3 ligation also induced CD3 co-clustering with CD8 in well-organized nano-domains of activated CD8 T cells; CD28 co-stimulation did not enhance CD8 clustering or CD3-CD8 co-clustering in nano-domains

**Figure 4:**
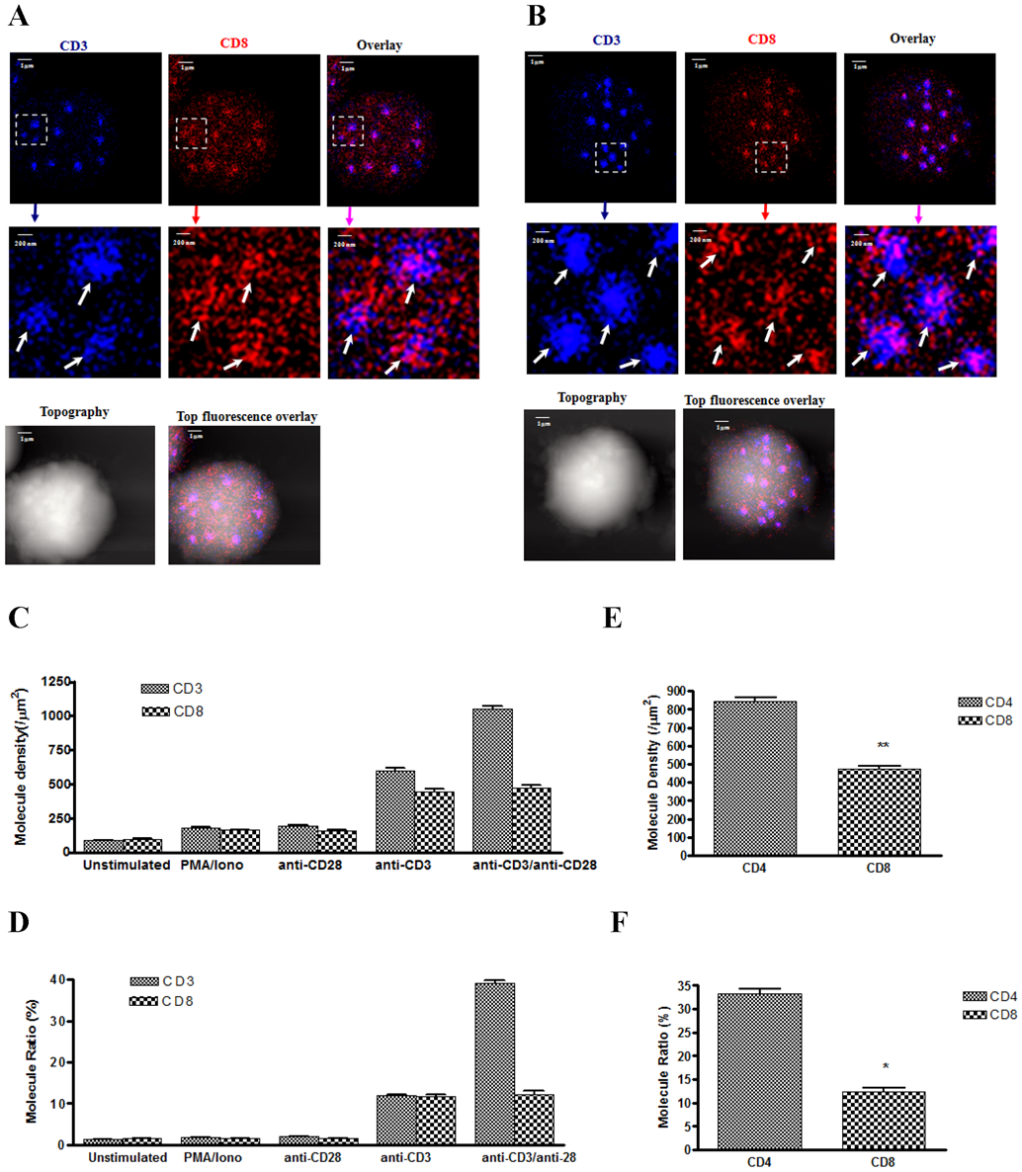
TCR/CD3 ligation also induced CD3 co-clustering with CD8 in well-organized nano- or micro-domains of activated CD8 T cells; CD28 co-stimulation did not enhance CD8 clustering or CD3-CD8 co-clustering in nano-domains. The scale bar for the middle panel is 200 nm. The integration time for all the images was 30 ms with 400*400 scanning lines. (A) The NSOM dual color images of one representative of the anti-CD3 Ab-stimulated CD8 T-cells. Note that both CD3 and CD8 formed nano-domains that were hardly seen in un-stimulated T cells. Nano-spatial co-clustering or co-localization of CD3 and CD8 (overlay pink color) was also seen in these nano-domains. Lower panels show the T cell topography and topography-fluorescence overlay images. (B) The NSOM dual color images of one representative of the CD8 T-cells co-stimulated with anti-CD3 Ab and anti-CD28 Ab. Note that CD28 co-stimulation did not enhance CD8 clustering or CD3-CD8 co-clustering in nano-domains, although it increased molecular number and density of CD3 clustering in enlarged nano-domains. Lower panels show the T cell topography and topography-fluorescence overlay images. (C) Molecular-density analysis shows that anti-CD3 Ab-stimulated CD8 T cells exhibited significantly-greater molecular density of CD3 or CD8 in the nano- or micro-domains compared to un-stimulated T cells, PMA/Ionomycin-stimulated T-cells or anti-CD28 Ab only-stimulated T cells (*p*<0.05); Anti-CD28/anti-CD3 Ab co-stimulation significantly increases density of CD3 (*p*<0.05), but CD8 remains almost the same in the co-localized nano- or micro-domains compared to anti-CD3 Ab stimulation only. Up to 10 cells were analyzed. (D) Molecular-number analysis shows that anti-CD3 Ab-stimulated CD8 T cells exhibited significantly-greater percentage numbers of total CD3 or CD8 molecules that arrayed to form nano- or micro-domains compared to un-stimulated T cells, PMA/Ionomycin-stimulated T-cells or anti-CD28 Ab only-stimulated T cells (*p*<0.05); Anti-CD28/anti-CD3 Ab co-stimulation significantly increases numbers of CD3 (*p*<0.05), but co-localized CD8 remains almost the same in the co-localized nano- or micro-domains compared to anti-CD3 Ab stimulation only. Up to 10 cells were analyzed. Similar results were observed regardless of whether CD3 and CD8 were stained with QD605 or QD655. (E) Molecule density of CD4 in the nano- or micro-domains in the CD4 T cells co-stimulated with anti-CD3 and anti-CD28 Ab was much greater than CD8 in the CD8 T cells co-stimulated similarly (** indicates *p*<0.05). Up to 10 cells were analyzed. (F)The percentage numbers of CD4 molecules that arrayed to form nano- or micro-domains in the CD4 T cells co-stimulated with anti-CD3 and anti-CD28 Ab was much higher than CD8 in the CD8 T cells co-stimulated similarly (* indicates *p*<0.05). Up to 10 cells were analyzed.

Although both CD4 and CD8 function as co-receptors during T-cell signaling and activation, accumulating evidence suggests that CD8 may differ from CD4 in facilitating T-cell signaling and activation [41], [42], [43]. From the nanosclae imaging standpoint, we sought to determine whether TCR/CD3 ligation also led to CD3-CD8 co-clustering in the same nano- or micro-domains, and whether CD28 co-activation could also enhance CD3-CD8 co-clustering as seen for CD3-CD4 co-clustering. After anti-CD3 Ab stimulation of CD8 T cells, ∼65% of CD3 and CD8 molecules underwent nano-spatial re-distribution to array and cluster forming nano-domains (Fig. 4A, 4D, Figure S6A, Figure S6B). Such re-distribution and clustering led to increase molecular density in the CD3 and CD8 nano-domains, respectively, although CD8 nano-domains were smaller and less dense than CD3 nano-domains (Fig. 4A). The overlay NSOM images indicated that the TCR ligation indeed also induced CD3 co-clustering with CD8 co-receptor, despite that CD3 was much more dominant than CD8 in the same nano- or micro-domains after TCR/CD3 ligation (Fig. 4A). It was noted that co-clustering of CD3/CD8 induced by anti-CD3 simulation was abrogated significantly by pre-treatment of T cell by LCK inhibitor, PP2 (Supporting information, Figure S4B).

Surprisingly, CD28 co-stimulation did not enhance CD8 clustering or CD3-CD8 co-clustering in nano-domains (Fig. 4B, Figure S6C, Figure S6D), although it increased molecular number and density of CD3 clustering in enlarged nano-domains. After anti-CD3 and anti-CD28 co-stimulation, the average molecule density of CD3 increased to 1085±31/µm^2^, which was much higher than that observed in the T cells stimulated with the anti-CD3 alone (Fig. 4B, 4C, *p*<0.05). Since CD28 on CD8+ T cells was similarly expressed as CD4+ T cells (data not shown), the absence of significant CD3-CD8 co-clustering upon CD28 co-stimulation did not appear to be attributed to no or less expression of CD28 on CD8+ T cells. Consistently, the CD28 co-stimulation led to increased expression of IFN-γ by CD3 Ab-activated T cells (data not shown here). In contrast, the density of CD8 was only 482±21/µm^2^, quite similar to what was seen in the CD8 T cells activated with ant-CD3 Ab alone. Almost 3 CD3 versus 1 CD8 co-clustered together in each of the co-localized nano- or micro-domains after CD28 co-stimulation (Fig. 4D). In fact, the percentage of CD8 recruited to the nano- or micro-domains and CD8 molecular density in the nano- or micro-domains were much lower than their CD4 counterparts after anti-CD3 Ab and anti-CD28 Ab co-stimulation (Fig. 4E, 4F, *p*<0.05). It is also worth to point out that no CD3 co-clustering with CD8 in nano- or micro-domains in the membrane of T cells activated by anti-CD28 Ab alone or PMA/Ionomycin alone (Supplemental information, Figure S7Ai, Figure S7Aii, Figure S7Aiii, Figure S7Bi, Figure S7Bii, Figure S7Biii). Thus, similar to what was seen for CD4 T cells, TCR/CD3 ligation induced CD3 co-clustering with CD8 in well-organized nano- or micro-domains of activated CD8 T cells. However, CD28 co-stimulation did not enhance CD8 clustering or CD3-CD8 co-clustering in nano-domains although it increased molecular number and density of CD3 clustering in enlarged nano-domains.

## Discussion

The NSOM/QD-based imaging system directly images the nano-spatial relationship between TCR/CD3 and CD4 or CD8 co-receptor before and after activation of un-stimulated primary T cells. While flow cytometry, fluorescence and confocal mircoscapy show high-density, un-distinguished “dispersed caps” of TCR/CD3 [24], CD4 or CD8 on membrane of resting/un-stimulated αβ T cells, there is actually no nanoscale information conceiving whether and how each of these major molecules distribute and share nano-space on membrane. Now, the NSOM/QD-based nanoscale imaging system allows us to directly visualize distribution and organization of these molecules on T-cell membrane, since the NSOM/QD-based imaging is highly reproducible, with up to 10^4^ TCR/cell detectable [24]. To our surprise, only about 5–8% of CD3 receptor and CD4 or 10% CD8 co-receptor were expressed as single QD-bound molecules on membrane. Using the standard staining reagents and protocol [1∶1 dominant binding mode for Ab/streptavidin binding to ligand] [24], single QD fluorescence dots would be considered one or more than one CD3, CD4 or CD8 molecules. Most of CD3, CD4 or CD8 on membrane are clusters equivalent to two more QD fluorescence intensity. While these low-density nanoclusters may represent natural random distribution patterns or the pre-clustering of CD3/TCR [31], they might be caused by some uncontrolled factors such as thermic shock or isolation procedures. However, ≥40–50 nm nanospace conferring distinction among CD3 molecules or between CD3 and CD4 or CD8 co-receptor is clearly seen under the NSOM. NSOM/QD-based imaging appears to confer better resolution imaging for horizontal scanning of cell-surface molecules than total internal reflection fluorescence (TIRF) microscopy, although the latter is advantageous for real time dynamics [34], [44], [45], [46].

The nanoscale CD3 co-clustering with CD4 or CD8 in the co-localized nano-domains or micro-domains during CD3-induced and CD28-enhanced T cell activation may represent interaction between TCR/CD3 and CD4 or CD8 co-receptor. These co-clustering CD3 and CD4 or CD8 molecules are at least <40–50 nm proximity to each other in the clusters of nano-domains, as NSOM is capable to measure molecule sizes down to ∼50 nm [24] and to spatially resolve molecules that are of 40–50 nm distance. The nanoscale co-clustering of CD3 and CD4 or CD8 not only occurs and but also forms the co-localized nano- or micro-domains in the sustained T cell activation. Such nanoscale co-clustering or interaction between CD3 and CD4 or CD8 co-receptor is an active process linked to TCR/CD3 activation pathway rather than a passive event simply caused by activation-induced changes in cellular skeleton [47]. This notion is supported by the finding that PMA/Ionomycin-induced activation or CD28 Ab alone stimulation does not induce CD3 co-clustering with CD4 or CD8 co-receptor. In addition, CD28-enhanced activation after anti-CD28 Ab co-stimulation induces greater CD3-CD4 co-clustering in the nano- or micro-domains but leaves no changes in CD8 co-clustering in the co-localized nano- or micro-domains. In fact, the nanoscale CD3 co-clustering with CD4 or CD8 in the co-localized nano-domains or micro-domains during sustained T cell activation is consistent with what is reported in biochemical studies suggesting that CD3 delta chains could establish a functional link with CD8 [14]. And this is also in agreement with FRET studies of transient CD3-CD4 or CD3-CD8 interaction in transfected T hybridoma cells [15], [48], but not in agreement with the report suggesting that CD4 initially co-localize with CD3 after TCR ligation but subsequently depart away from the CD3 cluster in single-molecule transfected cell model [11]. It is worth to mention that our current work focuses on nano-spatial aspects of CD3 co-clustering with co-receptor during sustained activation of primary CD4 and CD8 T cells after CD3 signaling and CD3/CD28 co-signaling, therefore complements the finding seen in CD3 and/or CD4/CD8 transfected cell models [11], [15], [48].

Our NSOM/QD imaging data suggest that nanoscale interaction between TCR/CD3 and CD4 or CD8 co-receptor is not purely a MHCp-dependent process, but also can be induced by the TCR/CD3 activation pathway itself and be driven by tyrosine phosphorylation of Lck. CD3 Ab-induced activation appears to drive the nanoscale co-clustering or interaction between CD3 and CD4 or CD8 co-receptor in the co-localized nano- or micro-domains. This intrinsic capability of TCR/CD3 activation pathway suggests that once TCR/CD3 activation is initiated, it may positively feedback and enhance Lck-dependent recruitment of CD4 or CD8 co-receptor for sustaining TCR/CD3-induced T cell activation. It is reasonable to presume that the more CD4 or CD8 co-receptor are recruited by TCR/CD3, the more phosphorylation of Lck-ZAP70 would be induced to amplify TCR/CD3 activation pathway.

One of the new and interesting observations in the current study is that CD28 co-stimulation can clearly enhance nanoscale co-clustering of CD3 with CD4 but not CD8, and form greater size and density of CD3-CD4-co-clustered nano-domains or micro-domains. While most studies done to date have showed that CD28 co-stimulation increases TCR/CD3 signaling and promote full T cell activation [49], [50], [51], the possibility that CD28-mediated enhancement of TCR/CD3 signaling and activation involves the augmented CD3 interaction with CD4 has not been addressed. Our NSOM/QD imaging data clearly show that CD28 co-activation enhances nanoscale co-clustering of CD3 and CD4 and augments CD3-induced T cell activation, which is characterized by increases in molecular density and sizes of co-clustering CD3 and CD4 nano-domains or micro-domains. These data suggest that CD28 co-signaling-enhanced T cell activation may involve the augmentation of CD3-CD4 interaction. It would be interesting to determine whether CD28-enhanced CD3-CD4 interaction involves T-cell cytoskeletal rearrangements [52]. It is also worth to note that CD28 co-stimulation has been shown to increase tyrosine phosphorylation of substrates and consumption of Lck [50], [53]. The increased tyrosine phosphorylation and Lck consumption after CD28 co-stimulation can be explained here by the CD28 co-stimulation-enhanced co-clustering of CD3 and CD4 in nanodoamins, as the increased co-clustering or interaction after CD28 co-activation would enable more CD4-associated Lck to proximate CD3 for ZAP70 phosphorylation and cellular activation.

Another interesting finding from our NSOM/QD imaging studies is the demonstration of a different nanoscale co-clustering pattern for CD3 and CD8 compared to CD3 and CD4 interaction. While CD3 Ab-induced activation leads to nanoscale co-clustering of CD3 and CD4 or CD3 and CD8 in nano- or micro-domains, CD8 clustering and CD3-CD8 co-clustering appear to be less dramatic than CD4 clustering and CD3-CD4 co-clustering, respectively (Fig. 2A, Fig. 4A, 4C, 4D). More importantly, CD28 co-stimulation does not enhance CD8 clustering or CD3-CD8 co-clustering in nano-domains. This is quite contrasted to the CD28 co-stimulation-mediated enhancement of CD4 clustering and CD3-CD4 co-clustering in enlarged nano-domains or micro-domains. Nevertheless, CD28 co-stimulation appears to enhance CD8 T cell activation as there are notable increases in sizes and density of nanoscale CD3 clustering and CD3-predominant nano-domains or micro-domains on membrane of CD8 T cells co-stimulated with anti-CD3 and anti-CD28 Ab. From a functional standpoint, these results suggest that CD8 may be more efficient than CD4 in facilitating CD3-induced signaling and activation after TCR ligation and anti-CD3 Ab and anti-CD28 Ab co-engagement. This is consistent with the reports describing that CD8+ T cells require much fewer MHCp molecules than CD4+ T cells do for full activation of T-cells [54], [55]. On the other hand, the data may reflect the structure constraint for CD8-Lck-CD3 association, as Lck prefers to bind to CD4 than CD8 [56].

## Materials and Methods

### Animals and reagents

Three Rhesus (Macaca macutum) macaques without any treatment or vaccination, 4 to 8 years old, (3–5 kg weight) were used for collection and isolation of T-cells from blood. The animal use was approved by UIC IACUC. Anti-CD3-coated or anti-CD3/anti-CD28-coated 96-well plates were from BD Biosciences. RPMI-1640 culture medium was obtained from GibcoBRL Corp. Rabbit anti-human CD3 was from Dako. Mouse anti-human CD4 or CD8 antibody were from BD Pharmagin. Biotinylated anti-mouse IgG was from Invitrogen. PMA and Ionomycin were from Sigma. Lck inhibitor PP2 was from Calbiochem. Anti-rabbit IgG (H+L)-conjugated quantum dot (QD) 605 and streptavidin-conjugated QD 655 were from Invitrogen as well. QDs were centrifuged and filtered as previously described to remove aggregates of QDs [24]. All these antibodies and QDs have been validated for use at our lab [24], [57], [58].

### Lymphocyte isolation, T-cell stimulation, and immune staining

Peripheral blood was collected from Rhesus monkeys as described above. Peripheral blood mononuclear cells (PBMC) were separated by Ficoll-Hypaque gradient centrifugation and washed with phosphate-buffered saline (PBS) as described by the previous reports of our lab [57]. For the anti-CD3 Ab stimulation, PBMC at a cell density of 2×10^5^ cells/ml were seeded into anti-CD3 Ab-coated 96-well plates for 8-hours culture in RPMI 1640 containing 10% FBS at 37°C in a 5% CO_2_ atmosphere. For the anti-CD3/anti-CD28 Ab co-stimulation, PBMC at a cell density of 2×10^5^ cells/ml were seeded onto anti-CD3 Ab-coated 96-well plates and co-cultured with 5 ng/ml anti-CD28 Ab for eight hours in RPMI 1640 containing 10% FBS at 37°C in a 5% CO_2_ atmosphere. For anti-CD28 Ab only stimulation, PBMC at a cell density of 2×10^5^ cells/ml were seeded into 96-well plates and co-cultured with 5 ng/ml anti-CD28 for eight hours in RPMI 1640 containing 10% FBS at 37°C in a 5% CO_2_ atmosphere. For PMA/Ionomycin stimulation, PBMC at a cell density of 2×10^5^ cells/ml were seeded into 96-well plates and co-cultured with 10 ng/ml PMA and 1 µ Ionomycin for 8-hours in RPMI 1640 containing 10% FBS at 37°C in a 5% CO_2_ atmosphere. To determine if tyrosine phospholation of Lck involved the intrinsic CD3 co-clustering with CD4 or CD8, T cells were pre-treated for 30 minutes with Lck inhibitor PP2 (0.1 mg/ml), and then were stimulated with anti-CD3 Ab as decribed above.

For T-cell immunolabeling, 2% formalin/PBS solution was first used to fix T-cells for 20 mins to rule out the possibility of non-specific activation of T-cells which induced by antibody labeling [24]. Our repeated experiments showed that formalin fixation followed by antibody staining did not induce artificial nanostructures of T cells (data not shown). In the first color labeling, purified mouse anti-human CD4 or CD8 antibody were used to label corresponding molecules, respectively, followed by biotinylated anti-mouse IgG conjugate to CD4 or CD8 antibody, then QD streptavidin conjugated 655 to conjugate to IgG. In the second color labeling, rabbit anti-human CD3 was used to label CD3, followed by anti-rabbit QD IgG (H+L) conjugated 605. Finally, 2% formalin/PBS solution was used again to further fix the cells. For all of above each labeling step, FBS/PBS was applied to wash twice to remove any unbound antibody or QDs. For NSOM imaging study, dd water suspensions of cells were spread onto glass cover slides that were pretreated with poly-L-lysine (Sigma) and air-dried at room temperature for NSOM imaging. Non-specific staining was not seen under the NSOM for the controls using isotype control antibody followed by immune-conjugated QD or QD alone, as described previously [24]. Non-specific staining was not seen either when anti-human TCR Ab was used to stain mouse T cells (data not shown). Ab- or streptavidin-conjugated QD appear to have sizes of ≈25 nm [24]. (CD3 complex and CD4 or CD8 may have nano-spatial sizes of ∼10 nm and ∼5–8 nm respectively).

### NSOM imaging and polarization detection

An Aurora-3 NSOM system (Veeco) was used in this study. The system is shown schematically in our pervious study [25]. The continuous wave semiconductor laser (Coherent, USA; Cube, 404 nm) was launched into a single mode optical fiber (Thorlabs Inc, USA) and used as excitation source. Straight, aluminum -coated probe (Veeco) with an aperture diameter of 50–80 nm was used for imaging. It should be noted that no significant difference in full width at the half maximum (FWHM) of fluorescent spots when we used different probes [24]. The probe tip was attached to piezoelectric quartz tuning fork (resonance frequency ∼93 KHz), and probe-sample distance was maintained constant of 10 nanometers by tuning-fork-based shear-force feedback. This mode of operation provided simultaneous topographic and optical data, which was collected with a 40×, NA 0.65 objective (Olympus, Japan) and split into two beams by a polarizing cube beamsplitter (Newports Inc. USA), then detected by two APDs (PerkinElmer, Canada) in 0° and 90°, respectively. Optical filters 655±10 nm and 605±10 nm (Newports Inc. USA) were used to separate the fluorescence from the excitation light and the background. The samples were mounted onto the XY stage with full scanning range of 30 micro;m×30 µm, and a video camera was used to locate the regions of interested. The images were stable and reproducible during repeated scanning. In this study, the laser excitation intensity was 120 W/cm^2^, the images consisted of 400×400 measured points, and most images have been slightly low-pass filtered.

### Image processing, data analyses and statistics

SPMLab 6.02 software (Veeco) was used to obtain high quality NSOM fluorescence image by leveling and convolution. The color scale ranges from red to green, reflecting a 90° change in in-plane orientation. Mathlab7.0 were used to calculate the fluorescence intensity and measure FWHM distribution of fluorescent spots. The number of QD molecules in each fluorescence spot was estimated based on the fluorescence intensity of single QD (see below), wheareas the intensity of each spot was determined by adding all photon counts with a contour of 15% of the peak intensity. For the molecular density determination, the fluorescence intensity of fluorescent spots was analyzed to determine the average fluorescence signal representing the average QD numbers. At the excitation laser intensity of 120 W/µm^2^, a typical count rate for individual QD 655 and QD605 were ≈7,000 counts/second and ≈4,500 counts/second, respectively (these values were reproducible in repeat experiments). And then the QD numbers were used to correlate the molecule numbers based on the conservative assumption that the QD: secondary Ab: primary Ab: target molecule = 1∶1∶1∶1 [24], [25]. And then the molecular density was determined by dividing the molecule numbers over the nano- or micro-domains areas. Student *t*-test was used to calculate the *p*-value, as described previously [59], to determine the statistical difference of molecular density or the percentages of molecules that localized into nano- or micro- domains after different stimulations.

## Supporting Information

Figure S1 The NSOM/QD-based polarized imaging system indicated that CD3, CD4 or CD8 molecules were distinctly distributed as single QD-bound molecules or nano-clusters equivalent to two or more QD fluorescence intensity on cell-membrane of un-stimulated primary T cells For the NSOM/QD-based polarized imaging system, the fluorescence emission is split into two images with orthogonal polarization components by using a polarization-beam splitter (PBS). The red images are for parallel polarization component (0°); the green images are for vertical polarization component (90°). Upper panels in each of the sub-figures show fluorescence images of a whole cell; Middle panels show zoom images of the areas as indicated by the squares on the top panels. Lower panels show the T cell topography and topography-fluorescence overlay images. Scale bars are indicated in each sub-figure. The integration time for all the images was 30ms with 400*400 scanning lines. (Ai) Nanoscale polarized CD3 images of one representative of the un-stimulated CD4 T-cells. Note that only about 5–8% of CD3 were distributed as single-QD-bound molecules (illustrated by red or green circulated dots) since these individual QD dots were equivalent to fluorescence intensity and FWHM of one QD spot and detectable on either horizontal [red, (0°)] or vertical [green, (90°)] polarization component. The majority of QD-bound CD3 molecules were imaged in both 0° and 90° polarization components [yellow] and distributed as nano-clusters equivalent to two or more QD fluorescence intensity/FWHM on the cell-surface. (Aii) Two histograms show frequency distribution of fluorescence intensity (upper panel) and size (FWHM) (lower panel) of QD-bound CD3 on membrane of ten T cells. Single or multiple QD-bound CD3 were judged based on the single QD fluorescence intensity and size (FWHM) as previously published [1] as well as polarization detection. (Bi) Nanoscale polarized CD4 images of one representative of the un-stimulated CD4 T-cells. Legends are the same as Fig. 1A. About 5–8% of CD4 were truly single-QD-bound molecules, whereas majority of them were nano-clusters equivalent to two or more QD fluorescence intensity and FWHM. (Bii) Two histograms show frequency distribution of fluorescence intensity (upper panel) and size (FWHM) (lower panel) of QD-bound CD4 on membrane of ten T cells. (Ci) Nanoscale polarized CD8 images of one representative of the un-stimulated CD8 T-cells. Unlike CD3 or CD4, a little bid more than 10% of CD8 was single-QD-bound molecules, whereas others were nano-clusters equivalent to two or more QD fluorescence intensity and FWHM. (Cii) Two histograms show frequency distribution of fluorescence intensity (upper panel) and size (FWHM) (lower panel) of QD-bound CD8 on membrane of ten T cells. (D)NSOM/QD-based imaging suggested that no significant fluorescence was observed on T cells when we used QD-streptavidin only to stain cells. (E) NSOM/QD-based imaging suggested that no significant fluorescence was observed on T cells when we used antibody only to stain cells. References: 1. Zeng G, Chen J, Zhong L, Wang R, Jiang L, et al. (2009) NSOM- and AFM-based nanotechnology elucidates nano-structural and atomic-force features of a Y. pestis V immunogen-containing particle vaccine capable of eliciting robust response. Proteomics 9: 1538–1547.
(1.73 MB DOC)

Figure S2 (A) Two histograms show frequency distribution of fluorescence intensity (left panel) and size (FWHM) (right panel) of QD-bound CD3 on membrane of ten resting CD4 T cells. (B) Two histograms show frequency distribution of fluorescence intensity (left panel) and size (FWHM) (right panel) of QD-bound CD4 on membrane of ten resting CD4 T cells. (C) Two histograms show frequency distribution of fluorescence intensity (left panel) and size (FWHM) (right panel) of QD-bound CD3 on membrane of ten resting CD8 T cells. (D) Two histograms show frequency distribution of fluorescence intensity (left panel) and size (FWHM) (right panel) of QD-bound CD8 on membrane of ten resting CD8 T cells.
(0.06 MB PPT)

Figure S3 (A) Two histograms show frequency distribution of fluorescence intensity (left panel) and size (FWHM) (right panel) of QD-bound CD3 on membrane of ten anti-CD3 Ab-stimulated CD4 T cells. (B) Two histograms show frequency distribution of fluorescence intensity (left panel) and size (FWHM) (right panel) of QD-bound CD4 on membrane of ten anti-CD3 Ab-stimulated CD4 T cells. (C) Two histograms show frequency distribution of fluorescence intensity (left panel) and size (FWHM) (right panel) of QD-bound CD3 on membrane of ten PMA/Ionomycin-stimulated CD4 T cells. (D) Two histograms show frequency distribution of fluorescence intensity (left panel) and size (FWHM) (right panel) of QD-bound CD4 on membrane of ten PMA/Ionomycin-stimulated CD4 T cells.
(0.07 MB PPT)

Figure S4 Lck inhibition significantly inhibited the cluster formation of CD4(A) and CD8(B), respectively. The Lck inhibition was done by pre-treatment of T-cells with Lck inhibitor PP2 for 30 minutes. And these T-cells were stimulated with anti-CD3 antibody. Note that no significant clusters were observed for CD4 and CD8, respectively.
(1.33 MB PPT)

Figure S5 (A) Two histograms show frequency distribution of fluorescence intensity (left panel) and size (FWHM) (right panel) of QD-bound CD3 on membrane of anti-CD3/anti-CD28 co-stimulated CD4 T cells. (B) Two histograms show frequency distribution of fluorescence intensity (left panel) and size (FWHM) (right panel) of QD-bound CD4 on membrane of anti-CD3/anti-CD28 co-stimulated CD4 T cells. (C) Two histograms show frequency distribution of fluorescence intensity (left panel) and size (FWHM) (right panel) of QD-bound CD3 on membrane of ten anti-CD28-stimulated CD4 T cells. (D) Two histograms show frequency distribution of fluorescence intensity (left panel) and size (FWHM) (right panel) of QD-bound CD4 on membrane of ten anti-CD28-stimulated CD4 T cells.
(0.06 MB PPT)

Figure S6 (A) Two histograms show frequency distribution of fluorescence intensity (left panel) and size (FWHM) (right panel) of QD-bound CD3 on membrane of anti-CD3 co-stimulated CD8 T cells. (B) Two histograms show frequency distribution of fluorescence intensity (left panel) and size (FWHM) (right panel) of QD-bound CD8 on membrane of anti-CD3-stimulated CD8 T cells. (C) Two histograms show frequency distribution of fluorescence intensity (left panel) and size (FWHM) (right panel) of QD-bound CD3 on membrane of ten a anti-CD3/anti-CD28 co-stimulated CD8 T cells. (D) Two histograms show frequency distribution of fluorescence intensity (left panel) and size (FWHM) (right panel) of QD-bound CD8 on membrane of ten anti-CD3/anti-CD28 co-stimulated CD8 T cells.
(0.06 MB PPT)

Figure S7 The NSOM dual-color images show that CD28 co-stimulation or PMA/Ionomycin stimulation did not enhance CD8 clustering or CD3-CD8 co-clustering in nano-domains (A) The NSOM dual-color images of one representative of the anti-CD28 Ab only-stimulated T-cells. Anti-CD28 Ab stimulation alone did not enhance CD8 clustering or CD3-CD8 co-clustering in nano-domains, although it increased molecular number and density of CD3 clustering and enlarged nano-domains. Lower panels show the T cell topography and topography-fluorescence overlay images. (B) Two histograms show frequency distribution of fluorescence intensity (left panel) and size (FWHM) (right panel) of QD-bound CD3 on membrane of anti-CD28-stimulated CD8 T cells. (C) Two histograms show frequency distribution of fluorescence intensity (left panel) and size (FWHM) (right panel) of QD-bound CD8 on membrane of anti-CD28-stimulated CD8 T cells. (D) The NSOMdual-color images of one representative of the PMA/Ionomycin-stimulated T-cells. PMA/Ionomycin stimulation did not induce apparent co-localized CD3 and CD8 nano- or micro-domains on the membrane of PMA/Ionomycin-stimulated CD8 T-cells. Lower panels show the T cell topography and topography-fluorescence overlay images. (E) Two histograms show frequency distribution of fluorescence intensity (left panel) and size (FWHM) (right panel) of QD-bound CD3 on membrane of PMA/Ionomycin-stimulated CD8 T cells. (F) Two histograms show frequency distribution of fluorescence intensity (left panel) and size (FWHM) (right panel) of QD-bound CD8 on membrane of PMA/Ionomycin-stimulated CD8 T cells.
(0.71 MB PPT)
