# Correction: NSOM/QD-Based Direct Visualization of CD3-Induced and CD28-Enhanced Nanospatial Coclustering of TCR and Coreceptor in Nanodomains in T Cell Activation

**DOI:** 10.1371/annotation/0cc4d7c5-134f-4db0-919f-14f74dd7846e

**Published:** 2010-03-12

**Authors:** Liyun Zhong, Gucheng Zeng, Xiaoxu Lu, Richard C. Wang, Guangming Gong, Lin Yan, Dan Huang, Zheng W. Chen

In Figure S6, panels A and C are duplicates of panel C in Figure S5. Please view the correct Figure S6 here: 
