# Correction: NSOM/QD-Based Direct Visualization of CD3-Induced and CD28-Enhanced Nanospatial Coclustering of TCR and Coreceptor in Nanodomains in T Cell Activation

**DOI:** 10.1371/annotation/d1055919-c5cf-423b-a068-224c9eacc58a

**Published:** 2010-03-12

**Authors:** Liyun Zhong, Gucheng Zeng, Xiaoxu Lu, Richard C. Wang, Guangming Gong, Lin Yan, Dan Huang, Zheng W. Chen

In Figure S2, panel C is a duplicate of panel A. Please view the correct Figure S2 here: 
