# Correction: NSOM/QD-Based Direct Visualization of CD3-Induced and CD28-Enhanced Nanospatial Coclustering of TCR and Coreceptor in Nanodomains in T Cell Activation

**DOI:** 10.1371/annotation/5d0d3323-53ea-4e3a-a9f8-ca4cddcdce52

**Published:** 2010-03-12

**Authors:** Liyun Zhong, Gucheng Zeng, Xiaoxu Lu, Richard C. Wang, Guangming Gong, Lin Yan, Dan Huang, Zheng W. Chen

LZ should also be noted as having contributed to writing the paper. 

