# Supplementary figures and images for: NSOM/QD-Based Direct Visualization of CD3-Induced and CD28-Enhanced Nanospatial Coclustering of TCR and Coreceptor in Nanodomains in T Cell Activation
Source: PLoS One. 2009 Jun 17;4(6):e5945. doi: 10.1371/journal.pone.0005945 (PMC2693923; doi:10.1371/journal.pone.0005945)

## Slide 1
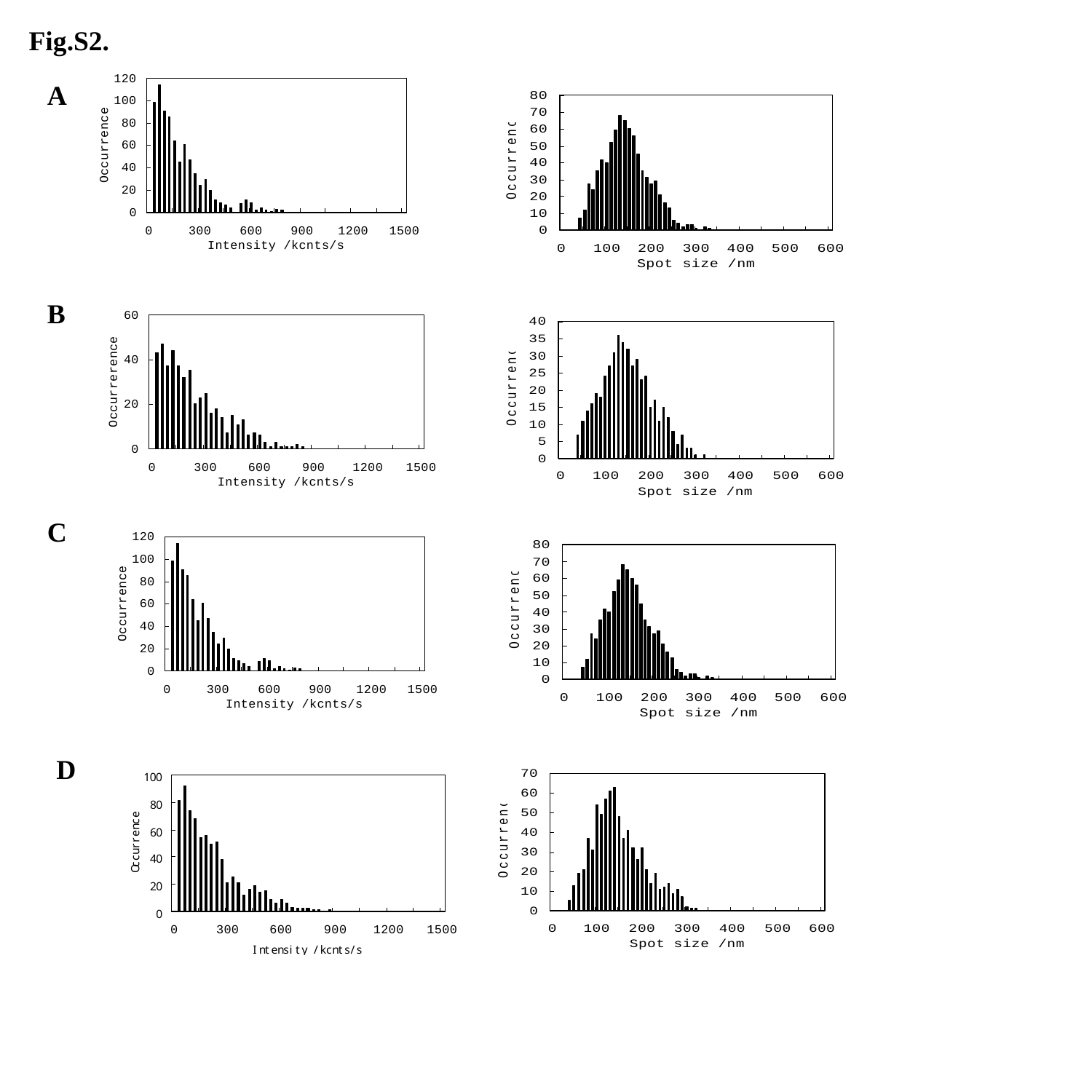

Fig.S2.
A
B
C
D

Supplement: Figure S2 — (A) Two histograms show frequency distribution of fluorescence intensity (left panel) and size (FWHM) (right panel) of QD-bound CD3 on membrane of ten resting CD4 T cells. (B) Two histograms show frequency distribution of fluorescence intensity (left panel) and size (FWHM) (right panel) of QD-bound CD4 on membrane of ten resting CD4 T cells. (C) Two histograms show frequency distribution of fluorescence intensity (left panel) and size (FWHM) (right panel) of QD-bound CD3 on membrane of ten resting CD8 T cells. (D) Two histograms show frequency distribution of fluorescence intensity (left panel) and size (FWHM) (right panel) of QD-bound CD8 on membrane of ten resting CD8 T cells. (0.06 MB PPT) [file pone.0005945.s002.ppt]

## Slide 1
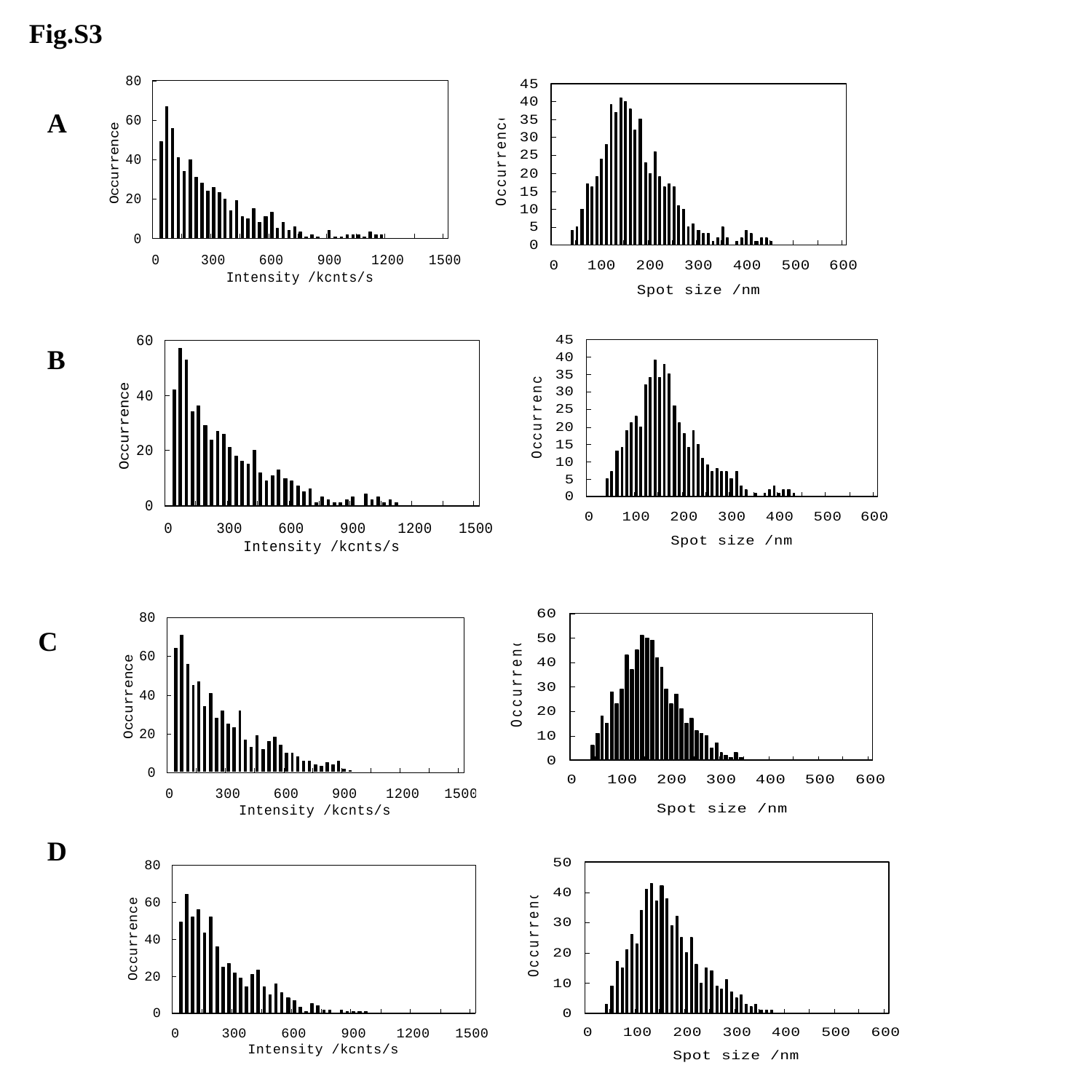

Fig.S3
A
B
C
D

Supplement: Figure S3 — (A) Two histograms show frequency distribution of fluorescence intensity (left panel) and size (FWHM) (right panel) of QD-bound CD3 on membrane of ten anti-CD3 Ab-stimulated CD4 T cells. (B) Two histograms show frequency distribution of fluorescence intensity (left panel) and size (FWHM) (right panel) of QD-bound CD4 on membrane of ten anti-CD3 Ab-stimulated CD4 T cells. (C) Two histograms show frequency distribution of fluorescence intensity (left panel) and size (FWHM) (right panel) of QD-bound CD3 on membrane of ten PMA/Ionomycin-stimulated CD4 T cells. (D) Two histograms show frequency distribution of fluorescence intensity (left panel) and size (FWHM) (right panel) of QD-bound CD4 on membrane of ten PMA/Ionomycin-stimulated CD4 T cells. (0.07 MB PPT) [file pone.0005945.s003.ppt]

## Slide 1
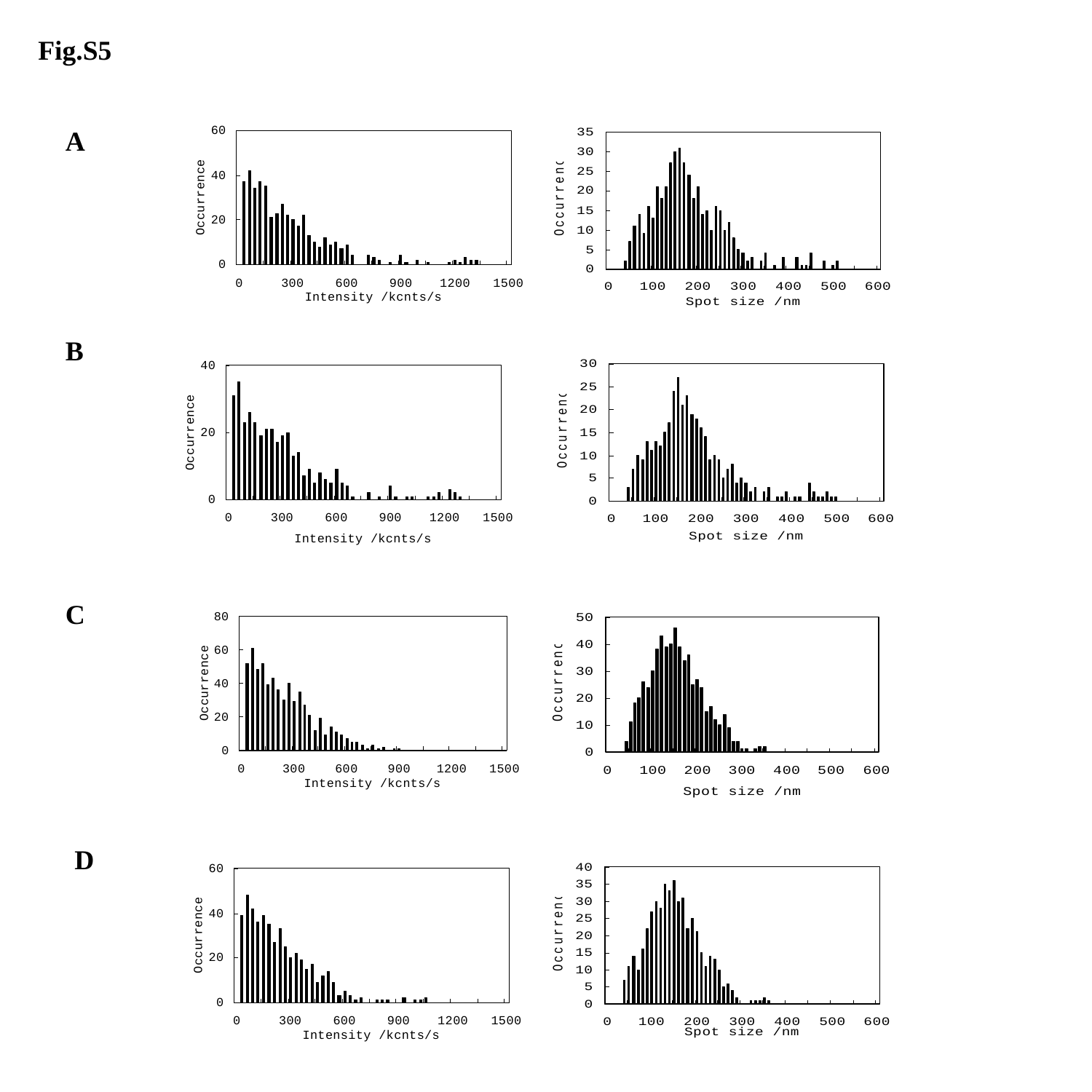

Fig.S5
A
B
C
D

Supplement: Figure S5 — (A) Two histograms show frequency distribution of fluorescence intensity (left panel) and size (FWHM) (right panel) of QD-bound CD3 on membrane of anti-CD3/anti-CD28 co-stimulated CD4 T cells. (B) Two histograms show frequency distribution of fluorescence intensity (left panel) and size (FWHM) (right panel) of QD-bound CD4 on membrane of anti-CD3/anti-CD28 co-stimulated CD4 T cells. (C) Two histograms show frequency distribution of fluorescence intensity (left panel) and size (FWHM) (right panel) of QD-bound CD3 on membrane of ten anti-CD28-stimulated CD4 T cells. (D) Two histograms show frequency distribution of fluorescence intensity (left panel) and size (FWHM) (right panel) of QD-bound CD4 on membrane of ten anti-CD28-stimulated CD4 T cells. (0.06 MB PPT) [file pone.0005945.s005.ppt]

## Slide 1
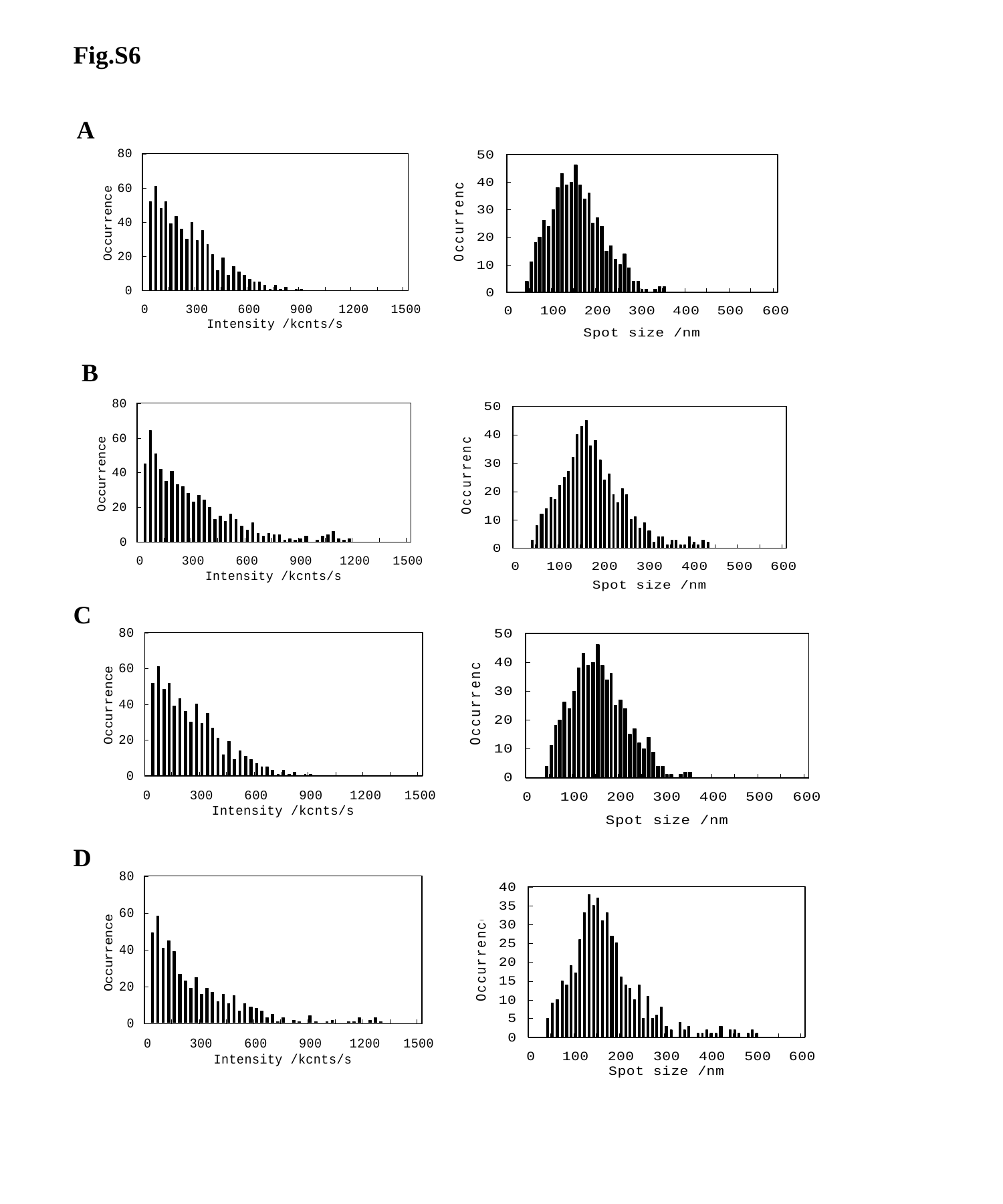

Fig.S6
A
B
C
D

Supplement: Figure S6 — (A) Two histograms show frequency distribution of fluorescence intensity (left panel) and size (FWHM) (right panel) of QD-bound CD3 on membrane of anti-CD3 co-stimulated CD8 T cells. (B) Two histograms show frequency distribution of fluorescence intensity (left panel) and size (FWHM) (right panel) of QD-bound CD8 on membrane of anti-CD3-stimulated CD8 T cells. (C) Two histograms show frequency distribution of fluorescence intensity (left panel) and size (FWHM) (right panel) of QD-bound CD3 on membrane of ten a anti-CD3/anti-CD28 co-stimulated CD8 T cells. (D) Two histograms show frequency distribution of fluorescence intensity (left panel) and size (FWHM) (right panel) of QD-bound CD8 on membrane of ten anti-CD3/anti-CD28 co-stimulated CD8 T cells. (0.06 MB PPT) [file pone.0005945.s006.ppt]

## Slide 1
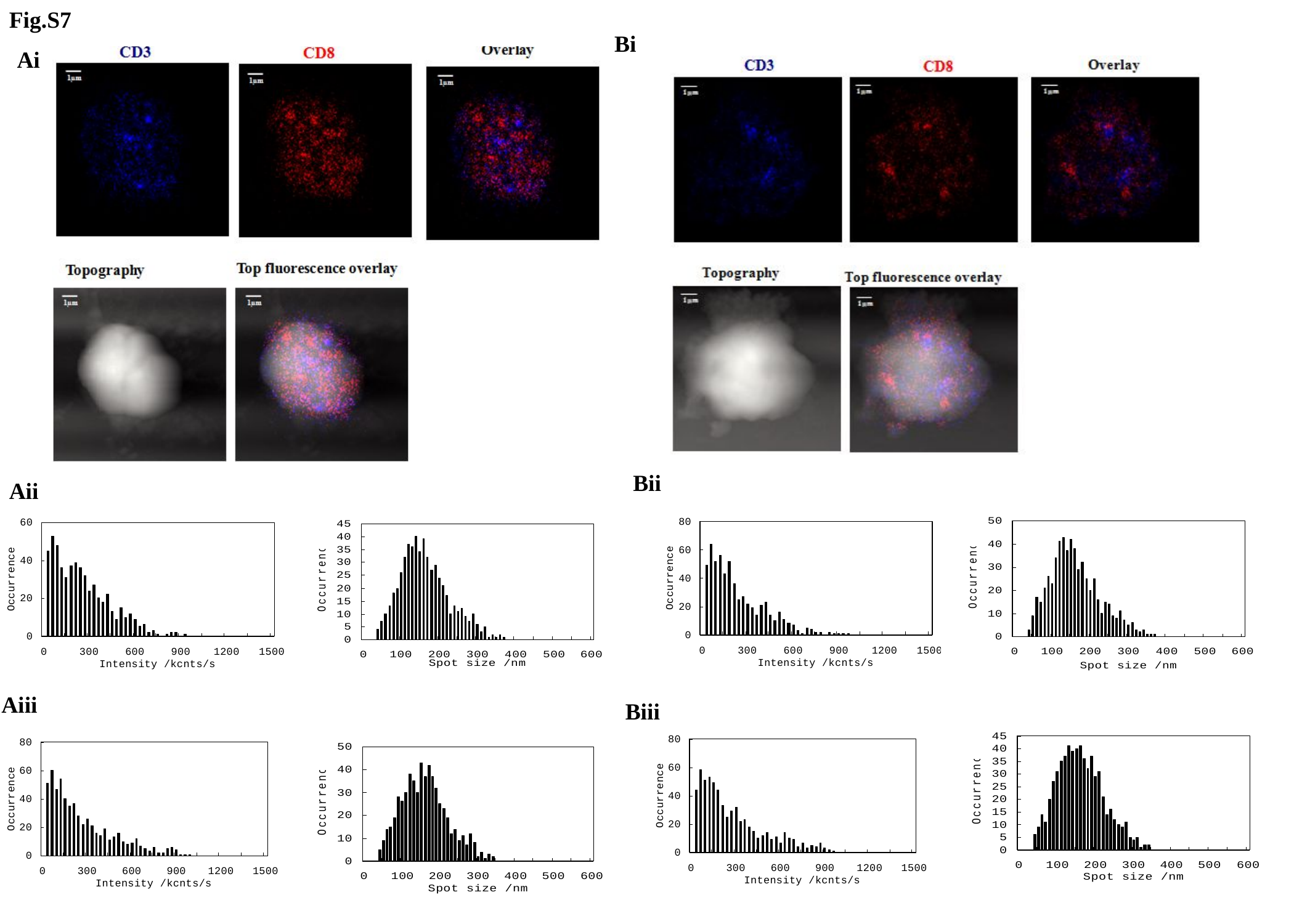

Fig.S7
Bi
Ai
Bii
Aii
Aiii
Biii

Supplement: Figure S7 — The NSOM dual-color images show that CD28 co-stimulation or PMA/Ionomycin stimulation did not enhance CD8 clustering or CD3-CD8 co-clustering in nano-domains (A) The NSOM dual-color images of one representative of the anti-CD28 Ab only-stimulated T-cells. Anti-CD28 Ab stimulation alone did not enhance CD8 clustering or CD3-CD8 co-clustering in nano-domains, although it increased molecular number and density of CD3 clustering and enlarged nano-domains. Lower panels show the T cell topography and topography-fluorescence overlay images. (B) Two histograms show frequency distribution of fluorescence intensity (left panel) and size (FWHM) (right panel) of QD-bound CD3 on membrane of anti-CD28-stimulated CD8 T cells. (C) Two histograms show frequency distribution of fluorescence intensity (left panel) and size (FWHM) (right panel) of QD-bound CD8 on membrane of anti-CD28-stimulated CD8 T cells. (D) The NSOMdual-color images of one representative of the PMA/Ionomycin-stimulated T-cells. PMA/Ionomycin stimulation did not induce apparent co-localized CD3 and CD8 nano- or micro-domains on the membrane of PMA/Ionomycin-stimulated CD8 T-cells. Lower panels show the T cell topography and topography-fluorescence overlay images. (E) Two histograms show frequency distribution of fluorescence intensity (left panel) and size (FWHM) (right panel) of QD-bound CD3 on membrane of PMA/Ionomycin-stimulated CD8 T cells. (F) Two histograms show frequency distribution of fluorescence intensity (left panel) and size (FWHM) (right panel) of QD-bound CD8 on membrane of PMA/Ionomycin-stimulated CD8 T cells. (0.71 MB PPT) [file pone.0005945.s007.ppt]
