# Supplementary material for: NSOM/QD-Based Direct Visualization of CD3-Induced and CD28-Enhanced Nanospatial Coclustering of TCR and Coreceptor in Nanodomains in T Cell Activation
Source: PLoS One. 2009 Jun 17;4(6):e5945. doi: 10.1371/journal.pone.0005945 (PMC2693923; doi:10.1371/journal.pone.0005945)
Supplement: Figure S4 — Lck inhibition significantly inhibited the cluster formation of CD4(A) and CD8(B), respectively. The Lck inhibition was done by pre-treatment of T-cells with Lck inhibitor PP2 for 30 minutes. And these T-cells were stimulated with anti-CD3 antibody. Note that no significant clusters were observed for CD4 and CD8, respectively. (1.33 MB PPT) [file pone.0005945.s004.ppt]

## Slide 1
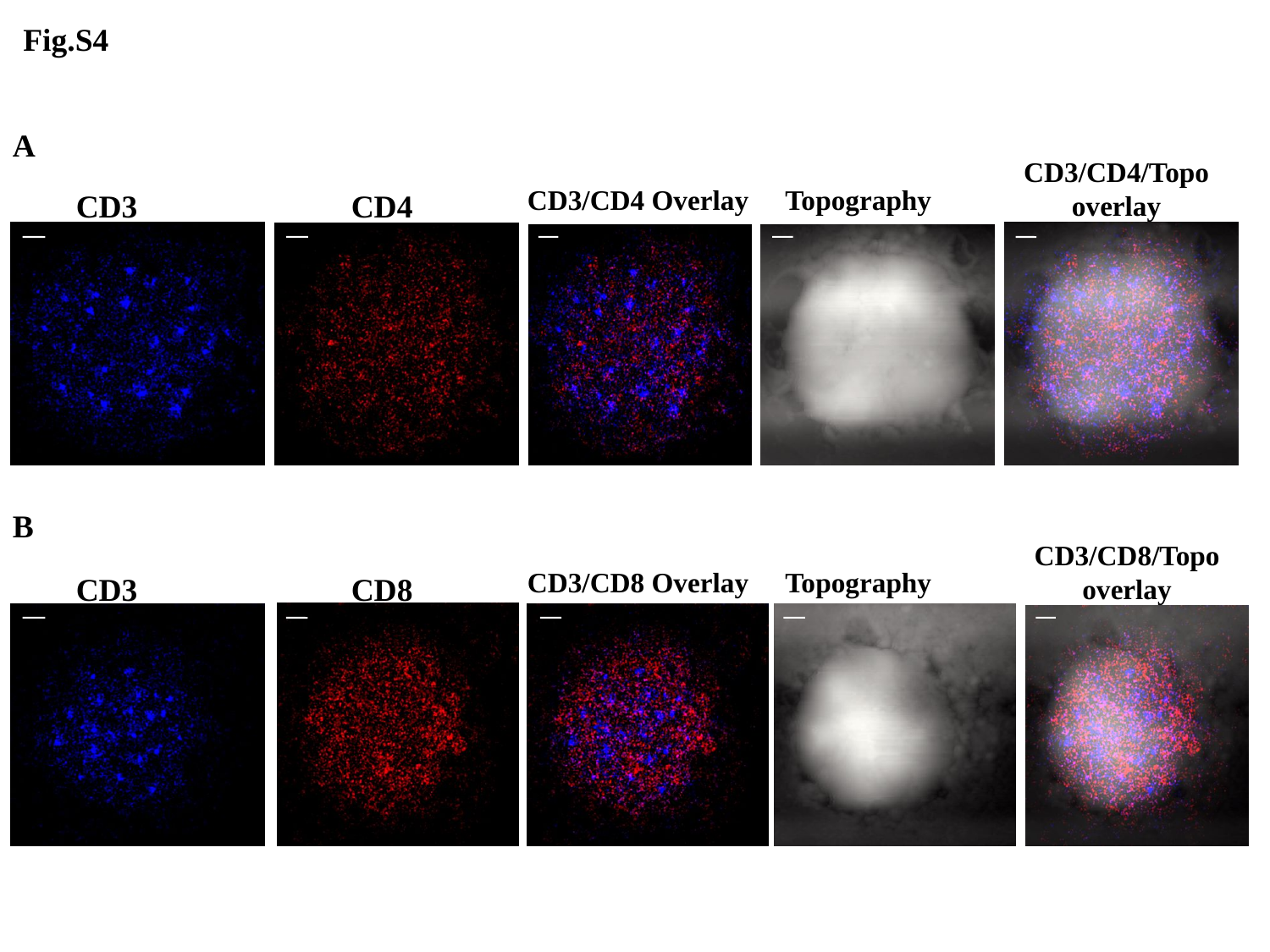

Fig.S4
A
CD3/CD4/Topo overlay
 CD3/CD4 Overlay
Topography
CD3
CD4
B
CD3/CD8/Topo overlay
 CD3/CD8 Overlay
Topography
CD3
CD8
