# Supplementary material for: NSOM/QD-Based Direct Visualization of CD3-Induced and CD28-Enhanced Nanospatial Coclustering of TCR and Coreceptor in Nanodomains in T Cell Activation
Source: PLoS One. 2009 Jun 17;4(6):e5945. doi: 10.1371/journal.pone.0005945 (PMC2693923; doi:10.1371/journal.pone.0005945)
Supplement: Supplementary file 3 [file pone.0005945.s001.doc]

Fig.S1

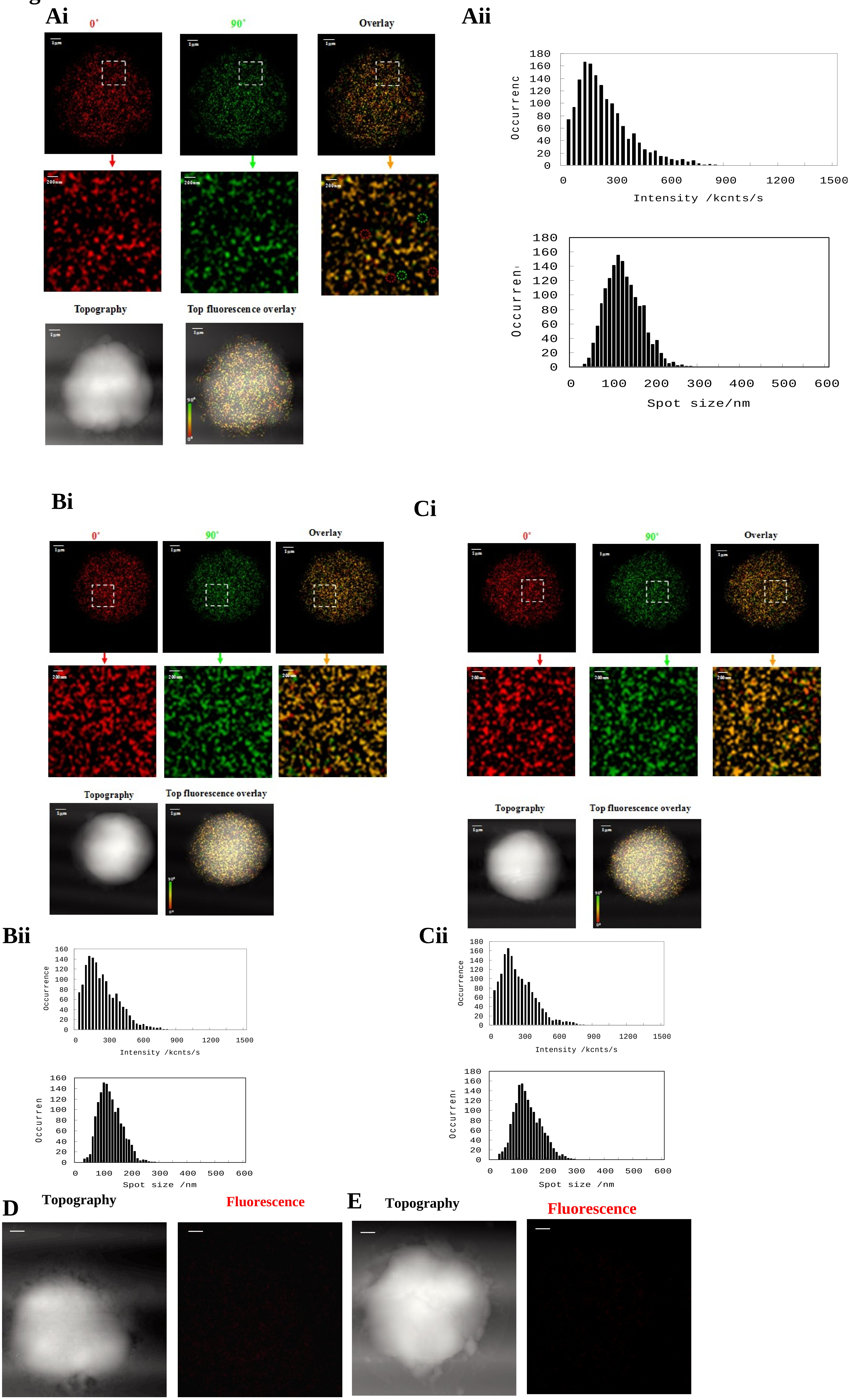

Ai
0°
90°
Overlay
1μm
200nm
Topography
Top fluorescence overlay
90°
0°
Aii
Occurrenc
Intensity /kcnts/s
0
300
600
900
1200
1500
180
160
140
120
100
80
60
40
20
0
Occurren
Spot size/nm
100
200
300
400
500
600
Bi
0°
90°
Overlay
Topography
Top fluorescence overlay
Ci
0°
90°
Overlay
Topography
Top fluorescence overlay
Bii
Occurrence
Intensity /kcnts/s
Occurren
Spot size /nm
Cii
Occurrence
Intensity /kcnts/s
Spot size /nm
D
Topography
Fluorescence
E
Topography
Fluorescence
