# Supplementary figures and images for: Correction: NSOM/QD-Based Direct Visualization of CD3-Induced and CD28-Enhanced Nanospatial Coclustering of TCR and Coreceptor in Nanodomains in T Cell Activation
Source: PLoS One. 2010 Mar 12;5(3):10.1371/annotation/0cc4d7c5-134f-4db0-919f-14f74dd7846e. doi: 10.1371/annotation/0cc4d7c5-134f-4db0-919f-14f74dd7846e (PMC2837715; doi:10.1371/annotation/0cc4d7c5-134f-4db0-919f-14f74dd7846e)

## Slide 1
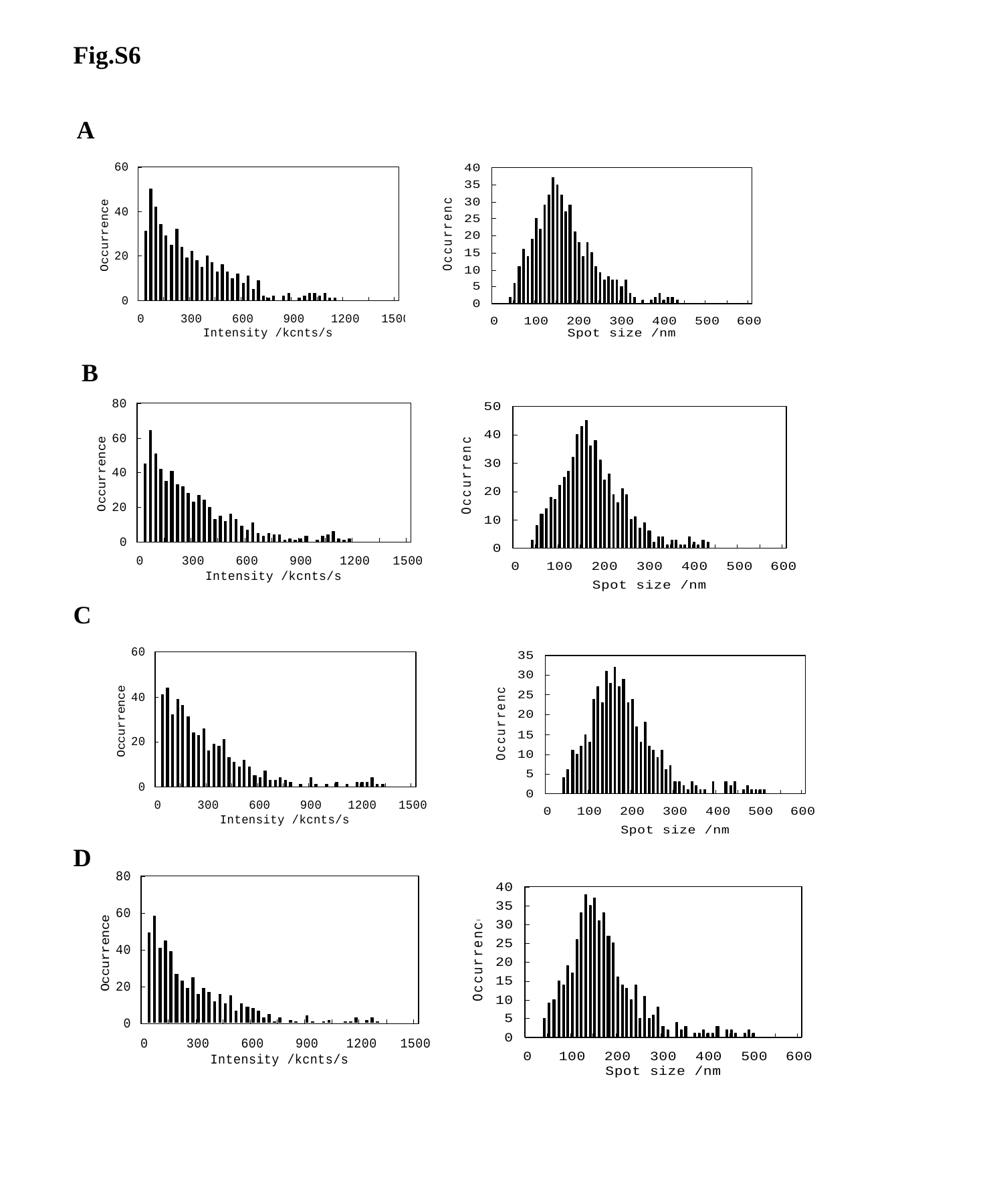

Fig.S6
A
B
C
D

Supplement: Supplementary file 1 [file pone.0cc4d7c5-134f-4db0-919f-14f74dd7846e.s001.ppt]
