# Supplementary figures and images for: Correction: NSOM/QD-Based Direct Visualization of CD3-Induced and CD28-Enhanced Nanospatial Coclustering of TCR and Coreceptor in Nanodomains in T Cell Activation
Source: PLoS One. 2010 Mar 12;5(3):10.1371/annotation/d1055919-c5cf-423b-a068-224c9eacc58a. doi: 10.1371/annotation/d1055919-c5cf-423b-a068-224c9eacc58a (PMC2837716; doi:10.1371/annotation/d1055919-c5cf-423b-a068-224c9eacc58a)

## Slide 1
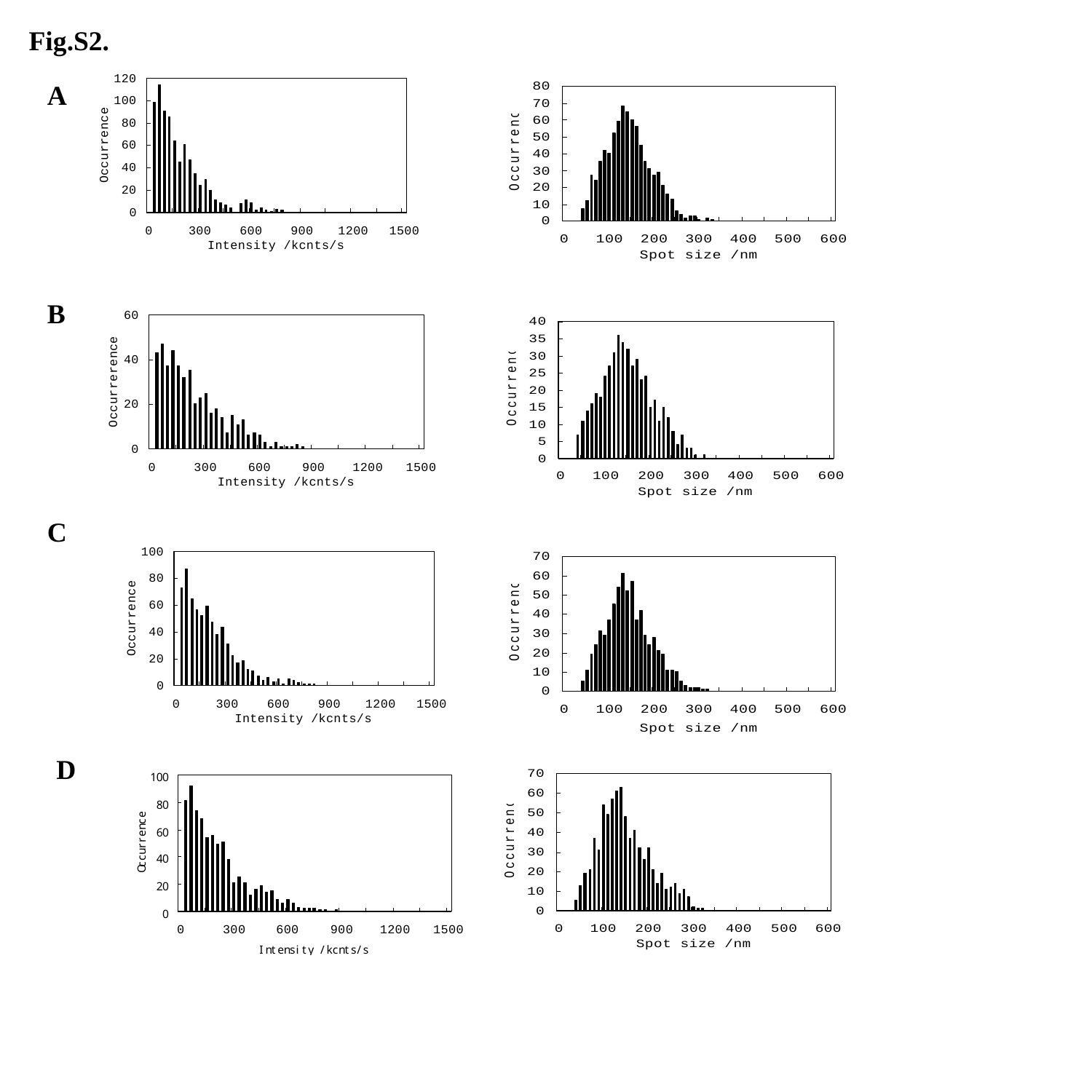

Fig.S2.
A
B
C
D

Supplement: Supplementary file 1 [file pone.d1055919-c5cf-423b-a068-224c9eacc58a.s001.ppt]
